# Calprotectin: two sides of the same coin

**DOI:** 10.1093/rheumatology/kead405

**Published:** 2023-08-21

**Authors:** Valeria Carnazzo, Serena Redi, Valerio Basile, Patrizia Natali, Francesca Gulli, Francesco Equitani, Mariapaola Marino, Umberto Basile

**Affiliations:** Department of Clinical Pathology, Santa Maria Goretti Hospital, AUSL Latina, Latina, Italy; Department of Clinical Pathology, Santa Maria Goretti Hospital, AUSL Latina, Latina, Italy; Facoltà di medicina e Chirurgia, Department of Clinical Pathology, Università “La Sapienza”, Rome, Italy; Clinical Pathology Unit and Cancer Biobank, Department of Research and Advanced Technologies, IRCCS Regina Elena National Cancer Institute, Rome, Italy; Department of Laboratory Medicine and Pathology, Azienda Ospedaliero Universitaria e Azienda Unità Sanitaria Locale di Modena, Modena, Italy; Clinical Biochemistry Laboratory, IRCCS “Bambino Gesù” Children’s Hospital, Rome, Italy; Department of Transfusion Medicine and Immuno-Hematology, Santa Maria Goretti Hospital, AUSL Latina, Latina, Italy; Dipartimento di Medicina e Chirurgia Traslazionale, Sezione di Patologia Generale, Università Cattolica del Sacro Cuore, Rome, Italy; Fondazione Policlinico Universitario “A. Gemelli” IRCCS, Rome, Italy; Department of Clinical Pathology, Santa Maria Goretti Hospital, AUSL Latina, Latina, Italy

**Keywords:** faecal calprotectin, serum calprotectin, IBD, autoimmune diseases

## Abstract

Calprotectin (CLP) is a calcium-binding protein produced by neutrophils and monocytes in the course of inflammation. Today, the role of faecal CLP in chronic IBD is well known, but in recent years attention has shifted towards circulating CLP. In fact, this molecule can be measured in different biological fluids: blood, saliva and urine, using different analytic methods that are described in this review. Furthermore, different data confirm the relevant role of serum CLP in autoimmune diseases. In this review we will highlight the correlation between high levels of circulating CLP and specific autoantibodies of major autoimmune pathologies paving the way to the employment of CLP measurement as useful biomarker for monitoring outcome in different pathologies.

Rheumatology key messages

- Calprotectin (CLP) is a protein secreted by activated monocytes and neutrophils in circulation during inflammatory processes.
- Serum CLP is a novel biomarker involved in the main autoimmune diseases.
- Recent studies highlight the importance of serum CLP as a therapeutic follow-up of many rheumatological diseases.

## Introduction

Calprotectin (CLP) is a soluble protein secreted by activated monocytes and neutrophils into the circulation, and is involved in inflammatory processes and/or inhibition of microbial growth.

**Figure 1.**
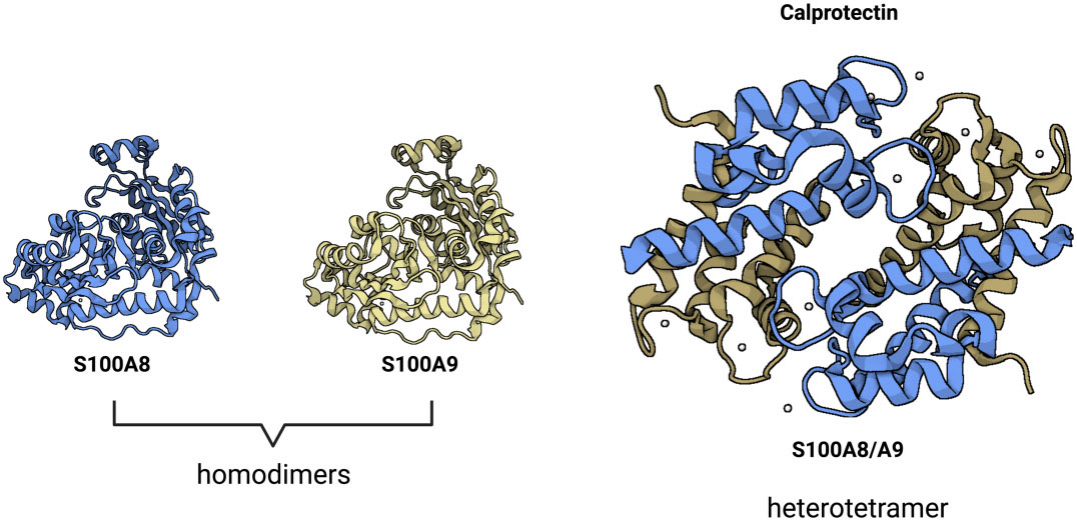
Structure of calprotectin (S100A8 and S100A9 proteins). S100A8 homodimer; individual subunits are shown in blue; S100A9 homodimer; subunits are shown in light brown; S100A8/A9 heterotetramer. Bound Ca^2+^ ions are shown by white spheres. Created with BioRender.com

CLP comprises a heterodimer belonging to calcium-binding protein of the S100 family and it is composed of two proteins named S100A8 and S100A9. In humans, these proteins are formed by two α-helix motifs that allow Ca^2+^ binding and other divalent metal ions such as Zn^2+^ [1]. After binding of the ion binding, the complex S100A8/S100A9 can form the heterodimer or heterotetramer that are considered essential to intracellular and extracellular biological function (Fig. 1). It has been observed that S100A8 and S100A9 can circulate as separated molecules, but the heterodimer is the most stable form, and it plays a key role in the protein’s biological interaction [2]. CLP can be assessed in faecal or serum samples. It has been described that CLP is mainly involved in inflammatory diseases. Faecal CLP (fCLP) is specific for gastrointestinal diseases while serum CLP (sCLP) is more specific in autoimmune diseases (AID) [3].

The serum level of CLP is usually reported below 1 μg/ml in healthy subjects but during inflammation the level may increase by 100 times. The faecal level of CLP (normal value up to 30 mg/l) provides a sensitivity and a specificity of 100% and 97%, respectively, in discriminating between active Crohn’s disease (CD) and irritable bowel syndrome (IBS) [4].

## Intra and extracellular function of calprotectin

**Figure 2.**
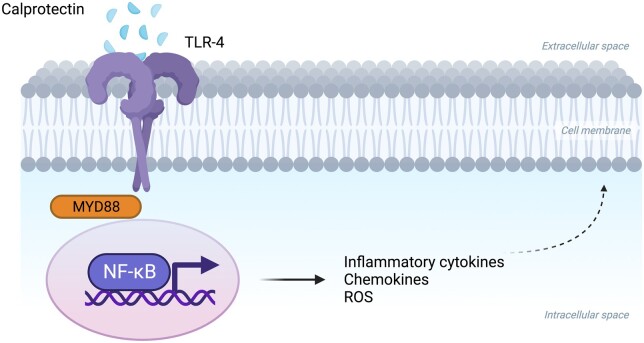
Extra and intracellular biological function of calprotectin. The extracellular function is mediated by Toll-like receptor 4 (TLR-4) which is the main calprotectin receptor. The bond between the heterodimer and TLR-4 triggers signal transduction cascade that involves nuclear factor-κB (NF- κB), which translocate into the nucleus and promote the expression of pro-inflammatory cytokines chemokines, and reactive oxygen species (ROS) that drive inflammation. Created with BioRender.com

CLP is involved in both intracellular and extracellular functions. In fact, the S100A8/S100A9 complex regulates intracellular pathways of immune cells and modulates inflammatory response. It allows leucocyte recruitment through leucocyte chemotaxis and tissue infiltration. The extracellular function is mediated by binding to receptors for advanced glycation end-products (RAGE) and Toll-like receptor 4 (TLR4) which is the main CLP receptor. The bond between the heterodimer and TLR4 triggers signal transduction cascade that involves nuclear factor-κB and MyD88, which translocate into the nucleus and promote the expression of pro-inflammatory cytokine genes such as *TNF-α*, *IL6*, *IL8*, *IL23*, etc. [5] (Fig. 2). Moreover, extracellular CLP complexes contribute to chelation of different transition metal ions which are important for bacteria.

## Faecal calprotectin

fCLP is a marker of intestinal inflammation. It is often used in children and adults for diagnosis of gastrointestinal diseases. In fact, fCLP can be used to distinguish IBD from other non-inflammatory bowel syndrome or to follow the progression of IBD in patients with established diagnosis [6].

## Application of faecal calprotectin

### Inflammatory bowel diseases

IBDs are a group of chronic, organ-specific, immune-mediated inflammatory diseases characterized by inflammation and damage to intestinal tract tissues. IBD has increasing incidence and prevalence in most countries and is becoming a global emerging disease. A westernized lifestyle or habits and some environmental factors have been found to contribute to the pathogenesis of IBD. The relevant risk factors include smoking, hygiene hypothesis, microorganisms, appendectomy, medication, nutrition and stress, which have all been found to be associated with the modality of IBD [7]. Females have a lower risk of CD compared with males until puberty, at which point there is a reversal, with females having a higher risk. Generally speaking, males and females demonstrate a similar incidence of ulcerative colitis (UC) before the age of 45 years; however, above age 45 years, males demonstrate a higher risk of incident UC than females [8].

The diseases pathways are not fully understood, but some IBDs could be associated with genetic predisposition, viruses and/or environmental factors showing autoimmune responses. The most common IBDs are CD and UC [9].

CD is a chronic inflammatory syndrome affecting the gastrointestinal tract and leading to extraintestinal complications [10].

CD can be located in different parts of gut [11], and based on this, the symptoms are:

- Ileum and colon—diarrhoea, cramping, abdominal pain, weight loss
- Colon only—diarrhoea, rectal bleeding, perirectal abscess, fistula, perirectal ulcer
- Small bowel only—diarrhoea, cramping, abdominal pain, weight loss
- Gastroduodenal region—anorexia, weight loss, nausea, vomiting

UC is a relapsing and remitting IBD of the large intestine. Diagnosis is suspected based on symptoms of urgency, tenesmus and haematochezia, and is confirmed with endoscopic findings of continuous inflammation from the rectum to more proximal colon, depending on the extent of disease. fCLP may be used to assess disease activity and relapse [12].

**Figure 3.**
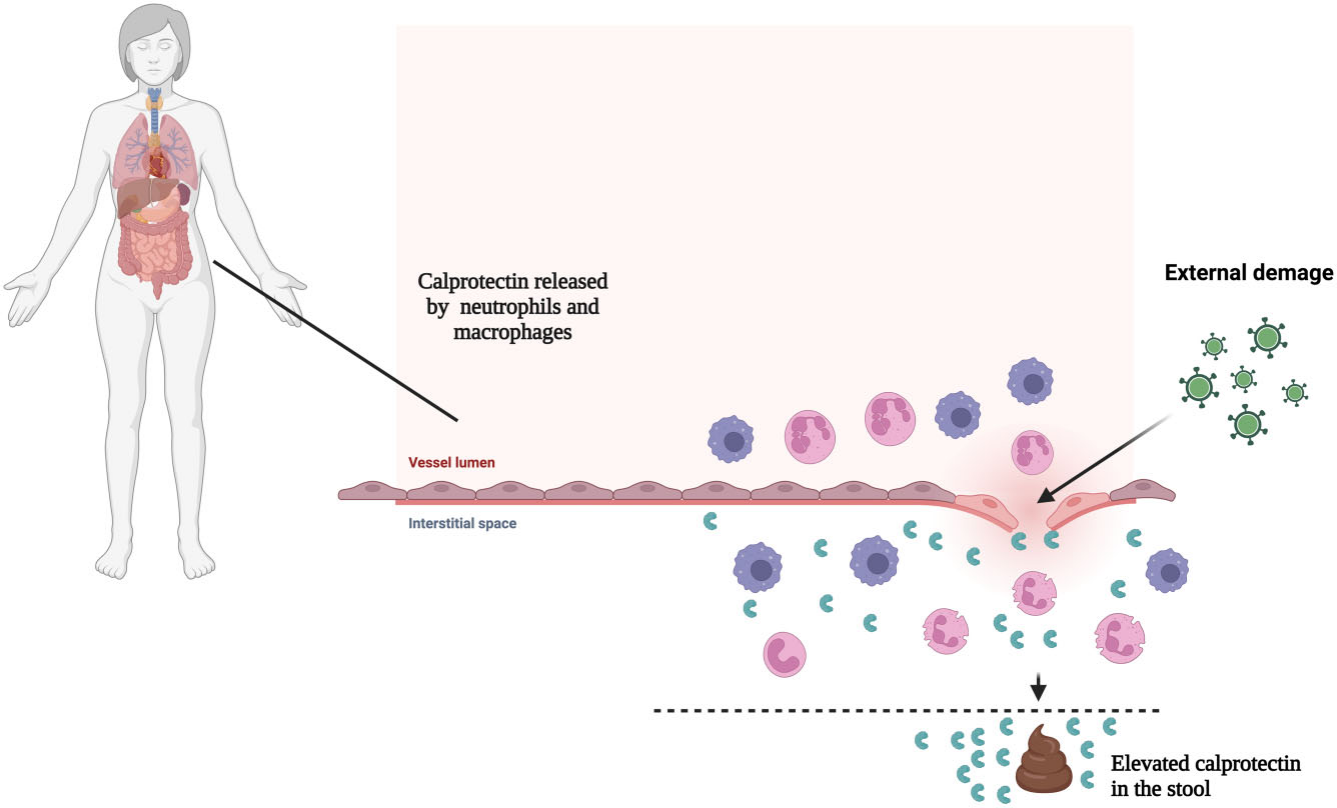
Calprotectin release by neutrophils and macrophages into intestinal space. After damage to the intestinal barrier, immune system cells are recruited into intestinal space and release calprotectin. This protein plays a key role to trigger inflammation. At the end, calprotectin is released through evacuation and it can be found in the stool of patients. Created with BioRender.com

Performing colonoscopy and histopathologic evaluation on an inflamed bowel biopsy specimen are currently considered as gold standards for the diagnosis and management of IBD. These techniques are known to be invasive and costly. In recent years, fCLP has received much attention for the diagnosis and non-invasive management of IBD. The presence of CLP in the faeces is directly proportional to neutrophil migration into the gastrointestinal tract during times of inflammation [13] (Fig. 3).

The fCLP test must be requested when the patient displays these symptoms: presence of blood in faecal material, diarrhoea, abnormal cramps, fever. The sample must not be contaminated by water or urine. It is often requested together with other stool tests such as ‘coprocultura’, which allows detection of bacterial infections, faecal leucocytosis and/or presence of ‘occult blood’ (faecal occult blood test). In addition, other tests to measure the inflammatory state may be required such as ESR and CRP. Other biological markers have been widely exploited in the last 10 years due to their availability, which also appear to be minimally invasive. They are employed in addition to endoscopic and histological procedures. Anti-*Saccharomyces cerevisiae* antibodies (ASCA) and perinuclear ANCA (pANCA) are routinely used to screen patients with clinical suspicion of IBD. ASCA are known to be predominantly associated with CD and ANCA with UC [14, 15]. All of these tests can be helpful as well to determinate the cause of the patient’s symptoms so as to exclude other disorders. A high level of fCLP can induce the clinician to request an endoscopic exam that is a confirmatory test for the diagnosis of IBD. However, due to its invasiveness, it is preferable not to perform it cases where there is not an inflammatory state. Measurement of fCLP may also be requested for a patient with IBD to monitor disease progression and assess disease severity. In fact, it is recommended to repeat the fCLP test after a few weeks in patients who have abnormal values [16].

In individuals with IBD, the level of fCLP can be very high (Table 1). Low concentrations usually correlate with a non-inflammatory disorder, such as viral infection or IBS. Rarely, IBS can provoke stomach cramps associated with diarrhoea but unlike IBD, these kinds of pathologies do not induce intestinal inflammation. For these reasons, in the presence of low levels of fCLP the patients are not referred for endoscopic examination. Variations in fCLP levels have been shown to correlate positively with age [20]. However, infants and children <10 years old have higher fCLP levels than adults [21].

**Table 1.** Sensibility and specificity of faecal calprotectin for the main intestinal inflammatory diseases

Disease	Value (cut-off)	Sensibility/specificity	Reference
CD	150 µg/g	85% sensibility/81% specificity	M.D. Jensen *et al.* [17]
UC	188 µg/g	98% sensibility/96% specificity	A.K. Jha *et al.* [18]
IBD	160 µg/g	100% sensibility/80% specificity	A. Diamanti *et al.* [19]

CD: Crohn’s disease; UC: ulcerative colitis.

### Detection and measurement of faecal calprotectin

The first method used to measure fCLP was described in the literature a long time ago. It comprised an ELISA that used a rabbit anti-CLP antibody. This assay has been shown to be associated with a relatively low yield of CLP and a high risk of contamination [22]. This issue was overcome by the development of a commercial assay that required 50/100 mg of faeces. The biological material is then dispensed into disposable capped tubes.

There are currently a wide range of commercial assays available for detection of fCLP [23], and the main ones are as follows: ThermoFisher EliA Calprotectin (fluorescence enzyme immunoassay (FEIA)), Eurospital Calprest (ELISA), Buhlmann (fCal ELISA), Diasorin Calprotectin (Chemi-Luminescence ImmunoAssay (CLIA)), Buhlmann fCal Turbo [particle enhanced turbidimetric immunoassay (PETIA)], Euroimmun Calprotectin (ELISA), Preventis GmbH PreventisID CalDetect (Lateral Flow Immunoassay (LFIA)), Buhlmann Labs AG Quantum Blue (LFIA), Organtec Calprotectin (ELISA), Calpro Inc. CalproLab (ELISA), Buhlmann Labs AG IBDoc (LFIA) and Calpro Inc. CalproSmart (LFIA).

All the mentioned kits involve immunochemical techniques using polyclonal or monoclonal antibodies that target different epitopes on the CLP molecule [24, 25].

The marked interassay variability has implications for the establishment of local laboratory cut-offs and requires those given in the literature to be interpreted appropriately [26]. This may not always be appreciated in the clinical setting; for example, a decrease in serial measurements performed in different hospitals might be interpreted as an initial response to treatment, but instead may merely reflect assay variability.

Prior to measurement, some sort of sample pretreatment is required to enable extraction of CLP from the faecal sample into a buffer for analysis. The most accurate method is to weigh out a small amount of faeces and add it to the buffer.

PhiCal (Calpro), an ELISA, was the first US Food and Drug Administration (FDA)–approved test for fCLP in the UDA, but the FDA has recently approved several newer fCLP assays for clinical use [27]. As shown in literature by Lin *et al*. [28], the comparison between PhiCal and four next-generation fCLP assays showed faster throughput using an automated immunoassay compared with an ELISA, and higher analytical ranges.

### Serum calprotectin

**Figure 4.**
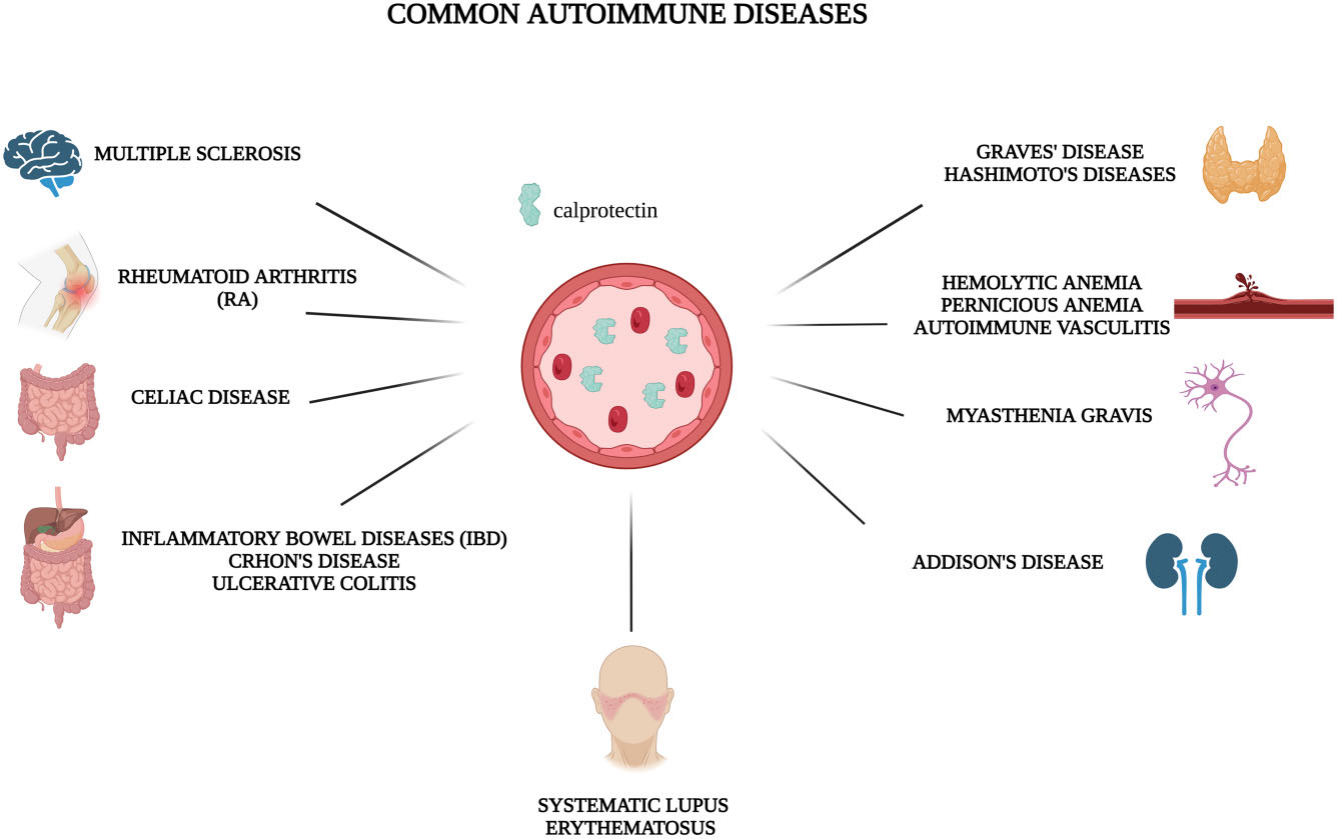
Serum calprotectinin common autoimmune diseases. Serum calprotectin levels could be a good alternative to acute-phase protein as a biomarker in the main autoimmune diseases. In fact, high levels of serum calprotectin are detected both in systemic autoimmune diseases such as RA, SLE, SS, SSc, myasthenia gravis and vasculitis, and in organ-specific autoimmune diseases as Hashimoto’s disease, coeliac disease, IBD and Addison’s disease. Furthermore, serum calprotectin levels are increased also in haemolytic autoimmune diseases as autoimmune haemolytic anaemia. Created with BioRender.com

sCLP levels could be a good alternative to acute-phase proteins as a biomarker in autoimmune diseases (AID) such as ESR, CRP, etc. The onset and development of AID are the consequence of interactions between genetic and environmental factors, which result in dysregulation of the immune system, characterized by the occurrence of autoantibodies and autoreactive T cells [29]. Based on this, circulating autoantibodies represent useful biomarkers of AID. These autoantibodies provide crucial diagnostic and prognostic information for the management of AID [30]. In particular settings, autoantibodies are not necessarily specific for AID. In fact, it has been observed that certain autoantibodies can also appear in the blood of healthy individuals [31] or in some particular physio-pathological situations, such as infections, the preclinical phase of AID or the administration of drugs [32]. It is noteworthy that the association between autoantibodies and risk of AID has attracted considerable attention. Numerous studies have shown that the presence of autoantibodies precedes the clinical onset of AID, and therefore could be a possible tool for AID screening or early diagnosis [33]. AID include a large spectrum of clinically distinct entities that share a common aetiology: a misguided, self-directed immune response. sCLP levels might be a good alternative to acute-phase protein as a biomarker in AID. In fact, high levels of sCLP are detected in both systemic AID such as RA [3], SLE, SSc, myasthenia gravis (MG) and vasculitis, and in organ-specific AID such as Hashimoto’s disease. Furthermore, sCLP levels are increased also in haemolytic AID such as autoimmune haemolytic anaemia (Fig. 4) [34].

### Role of serum calprotectin on the adaptive immune system

**Figure 5.**
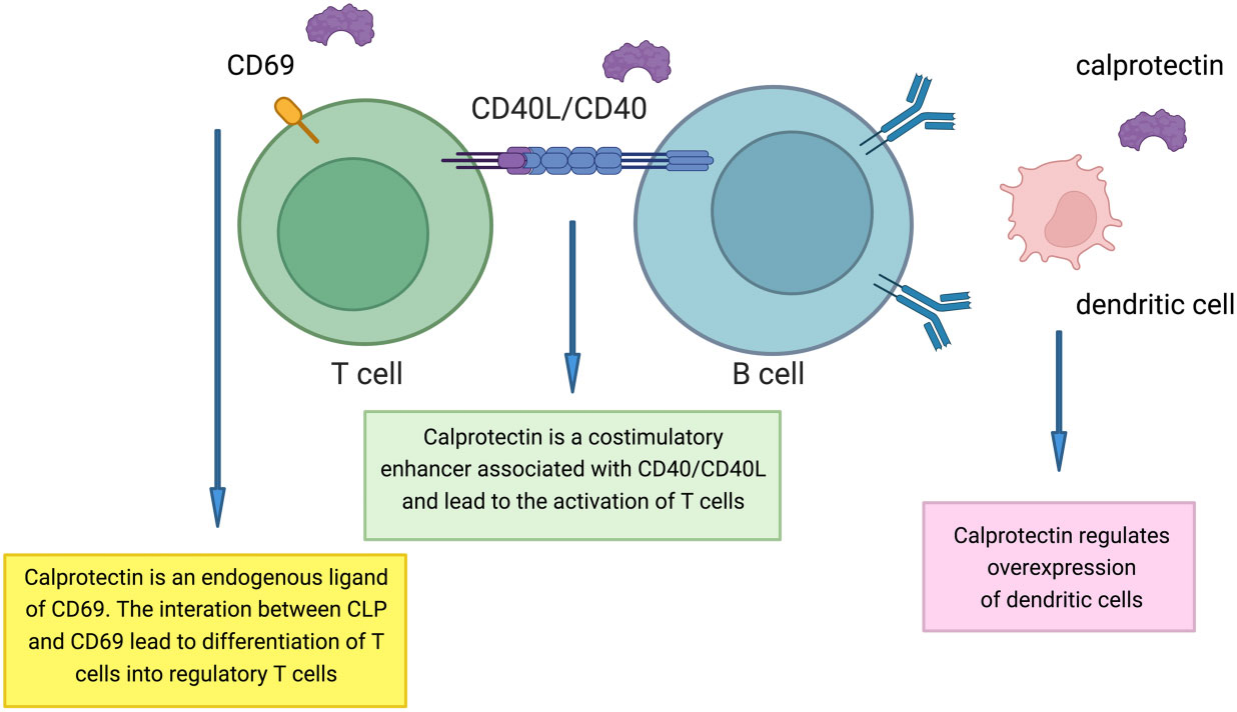
Role of serum calprotectin in autoimmune diseases. Calprotectin (CLP) may represent a connection between inflammation and the adaptive immune response. CLP contributes to the activation of CD8^+^ T cells during the process of activation by antigen-presenting cells via dendritic cell overexpression. CLP is an enhancer of co-stimulation of the CD40/CD40L signal leading to CD8^+^ T cell activation. Furthermore, CLP is an endogenous ligand of CD69. The interaction between CLP and CD69 leads to the differentiation of T cells into regulatory T cells. Created with BioRender.com

sCLP is an important inflammatory biomarker and plays a key role on the adaptive immune response. CLP contributes to the induction of CD8^+^ T cells during the activation process by antigen-presenting cells [35]. This protein is a costimulatory enhancer together with CD40/CD40 ligand signalling and leads to the loss of tolerance of T cells. In a murine model of autoimmunity, the absence of S100A8 and S100A9 resulted in reduced IL-17 production by autoreactive CD8^+^ T cells and in lower autoantibody production [35]. Given its low molecular weight (36.5 kDa), CLP may diffuse from inflamed tissues to the blood circulation (Fig. 5).

## Application of serum calprotectin

### Rheumatoid arthritis

In the literature, for patients affected by RA, a cut-off above 0.9 μg/ml was proposed to distinguish RA from non-inflammatory arthritis. In fact, sCLP levels are elevated in RA, but not in OA [36]. RA is the most common chronic inflammatory joint disease, characterized by the presence of RF and ACPA. Both ACPA and RF together with inflammatory markers such as ESR and CRP have been included in the current 2010 ACR/EULAR classification criteria for RA.

In one study [37], the authors highlighted the importance of sCLP in patients with RA who are receiving tocilizumab therapy to evaluate the inflammatory activity of the disease.

Approximately 140 patients were analysed, divided into two groups: patients receiving tocilizumab and patients receiving TNF inhibitor therapy. CLP, CRP and ESR were dosed on these patients. Level of sCLP increased in tocilizumab patients with active disease *vs* tocilizumab patients with no active disease, while CRP and ESR were unchanged. Conversely, in TNF-inhibitor patients with active disease the levels of three parameters are increased. CLP exhibits good sensitivity and specificity in patients with tocilizumab treatment for the detection of RA activity [37].

### Systemic lupus erythematosus

Circulating CLP also increases in SLE patients. SLE is a systemic AID characterized by the loss of self-tolerance and the production of anti-nuclear components autoantibodies. This condition causes systemic organ inflammations, including joints, kidneys and skin. However, the diagnosis of SLE is complex given that the pathophysiology is as yet unclear. Many studies have shown that serum CLP is high in patients with SLE compared with healthy controls. Furthermore, levels of CLP are associated with disease activity, high level of dsDNA antibodies and other SLE markers [38].

### Vasculitis

Other studies have investigated the role of CLP in vasculitis. Cutaneous and other vasculitides are specific inflammations of the blood vessel wall that can take place in any organ system of the body. Autoimmune vasculitis is a different group of diseases that cause damage to arteries, arterioles and capillaries. Vasculitis leads to destruction of the vascular wall resulting in haemorrhage and tissue ischaemia [39]. ANCA-associated vasculitis (AAV) is an AID characterized by serum-positive ANCA, better known as MPO- or PR3-AAV, and the rapidly progressive glomerulonephritis which shows pauci-immune complex deposition in pathogenic biopsy. It has been observed in several studies that high levels of CLP are correlated with high levels of MPO and PR3. The sCLP of active MPO-AAV significantly increased (compared with inactive AAV and healthy controls) and were correlated with the severity of the disease [40]. In remission phase, sCLP was higher than in healthy controls but lower compared with patients with acute state of disease [41].

### Systemic sclerosis

SSc is an AID characterized by fibrosis of skin and internal organs, vasculopathy, and dysregulation of immune system. A diagnostically important feature of immunological abnormalities in SSc is the presence of circulating ANA, which can be detected in 90–95% of patients. These include antibodies against topoisomerase (anti-TOPO I), kinetochore proteins (ACA), RNA polymerase enzyme (anti-RNAP III), ribonuclear proteins (anti-U11/U12 RNP, anti-U1 RNP, anti-U3 RNP) and nucleolar antigens (anti-Th/To, anti-NOR 90, anti-Ku, antiRuvBL1/2, anti-PM/Scl) [42]. SSc is an heterogeneous disease which leads to unclear diagnosis, and it is hard to predict its progression. For these reasons, it is necessary to study new biomarkers for diagnosis, progression and drug response.

### Sjögren’s syndrome

In many studies it has been shown that a high level of CLP is associated with mortality in SSc patients [43]. SS is a multifactorial systemic AID characterized by a wide spectrum of different clinical manifestations. The main symptoms are dry mouth and eyes. Conjunctivitis and periodontal disease, enlargement of the salivary glands, especially the parotid glands, dry cough, dry skin and dry genital organs. Other manifestations include arthralgias, paresthesias, asthenia and cutaneous vasculitis [44]. High levels of sCLP were associated with positive anti-SSA e anti-Ro60 with higher incidence of carotid atherosclerosis [45] (Table 2).

**Table 2.** Correlation between serum calprotectin and autoantibodies in different autoimmune diseases

Disease	Association with specific disease features	Prognosis
RA [46]	High levels are associated with positive RF and ACPA	High levels are predictive of disease relapse
SLE [38]	High levels associated with positive Ab-anti-dsDNA	High levels are predictive of structural damage (skin damage, glomerulonephritis)
SS [47]	High levels associated with positive anti-SSA and anti-Ro60	High levels associated with higher incidence of carotid atherosclerosis
SSc [42]	High levels are associated with positive Ab-anti-hystone, anti-U1RNP anti-Th/To, anti-NOR 90, anti-Ku, antiRuvBL1/2 and anti-PM/Scl70	High levels are predictive of reduced survival
Vasculitis [40]	High levels of CLP are correlated with high levels of MPO and PR3	High levels are predictive of disease relapse, associated with proliferative glomerulonephritis
Myasthenia gravis [48]	High levels of CLP are associated with presence of AchR-Ab, Musk-Ab and LRP4-Ab	High levels are correlated with severity of the disease
IBDs [49]	High levels of CLP are found in CD and UC. High levels are associated with positive ASCA and pANCA	High levels are found during active disease in CD but not in UC

CLP: calprotectin; CD: Crohn’s disease; UC: ulcerative colitis.

### Myasthenia gravis

MG is an AID characterized by an interruption in the transmission of contractile signals between nerves and muscles. The impairment of these stimuli causes the rapid onset of fatigue and intense muscle weakness. The disease is mediated by antibodies directed towards various proteins of the neuromuscular junction. In particular, 85% of myasthenic patients have antibodies targeting the muscle acetylcholine receptor (AChR); 6% have antibodies direct to muscle specific kinase (MuSK); and 1–3% have antibodies to low-density lipoprotein receptor-related protein 4 (LRP4). About 10% of MG patients have thymoma and other autoantibodies. The aetiology of MG and its heterogeneity in the clinical course are poorly understood. There is an urgent need for a sensitive biomarker in MG that reliably predicts individual disease course and exacerbation, as well as guiding immunosuppressive treatment. sCLP assay could be a potential biomarker in this pathology. In one study, the authors evaluated sCLP levels in MG patients and healthy controls and in different disease sets [48]. About 258 patients were evaluated, divided into subgroups according to age of onset. Patients were grouped into remission [Myasthenia Gravis Foundation of America (MGFA) 0], ocular (MGFA I) or generalized MG patients (MGFA II–IV). CLP levels are elevated in MG patients compared with healthy controls. Significantly higher CLP levels in patients with ocular and generalized MG than in patients in remission. This study demonstrates that CLP levels were significantly higher in MG than in controls. Furthermore, CLP may reflect disease severity.

### Detection and measurement of circulating calprotectin

sCLP is not as widely used as fCLP, but it has been found to be useful as an inflammatory marker and in particular is more specific for AID. However, blood CLP measurements can be affected by preanalytical variables such as type of anticoagulant, temperature and storage time. It has been seen in a study conducted by Kim *et al.* [38] that the detachment of CLP concentrations in particular conditions such as different anticoagulants and different temperatures, gives different results: CLP concentrations in EDTA samples stored for 2 h before analysis at 4°C, 20°C or 37°C did not differ (*P* = 0.15). Meanwhile, the concentrations of CLP in serum and Li-heparin are increased with increasing temperature, and the differences between all storage temperatures were significant for both anticoagulants (*P* < 0.001). CLP levels in EDTA tubes were significantly lower than CLP concentrations in serum at all temperatures. Furthermore, in this study, a significant linear correlation between serum CLP concentrations at 20°C and leucocyte count was highlighted. Meanwhile, there is a poor correlation of the protein concentrations in EDTA tubes. ELISA is the most used method for measuring CLP but it is usually associated with long test turnaround times. Another usable method is PETIA. These methods are usually used as a random-access test and the samples are run continuously as they arrive at the laboratory, which contributes to reduced test turnaround times [39]. ELISA kits are available with monoclonal as well as polyclonal antibodies. CLIA kits use a monoclonal antibody. Turbo assay is a PETIA using a polyclonal antibody reagent and it is commercialized as an open channel assay suitable for general clinical chemistry analyzers.

## Conclusion

Our literature highlights the role of fCLP and sCLP in different pathologies. Our data confirm the importance of fCLP, already widely used in laboratory diagnostics, for the management of IBDs and the emerging role of sCLP in AID. In fact, the presence of CLP in faeces reflects neutrophil migration into the gastrointestinal tract during inflammation and it is correlated with high levels of ESR and CRP. A high level of fCLP can prompt the clinician to request an endoscopic exam that is mandatory for the diagnosis of IBD. Low concentrations usually indicate a non-inflammatory disorder, such as viral infection or IBS, and rules out the patient from requiring endoscopic examination. Measurement of fCLP is useful in a patient with IBD to monitor disease progression and severity. Otherwise, high levels of sCLP have been found in the main autoimmune pathologies such as RA, SLE, SSc, MG and SS. A high concentration of this circulating molecule is associated with worse structural outcome but in addition it may predict disease relapse and severe manifestations of damage. For example, in AAV and SLE, high levels of sCLP correlate with glomerulonephritis and fibrosis. Furthermore, it has been observed that sCLP could be an excellent biomarker in MG; in fact, numerous studies confirm its usefulness in assessing the stage of the disease. Finally, sCLP is important in the evaluation of the inflammatory stage of patients with RA receiving tocilizumab therapy. sCLP is useful marker of inflammation compared with CRP and ESR in monitoring these patients. Our collection of literature shows the use of different dosage methods for both CLPs. We highlight the importance of sCLP in AID, especially with regard to systemic AID.

Based on the above, our work aims to highlight the importance of CLP in AID such as rheumatological ones. The use of sCLP in patients affected by rheumatological pathologies who do not show alterations in CRP and ESR could be useful for monitoring inflammation activity. Furthermore, we wish to highlight the role of sCLP in monitoring the clinical outcome and pharmacological follow-up in these diseases.

One of the problems still to be solved is the lack of standardization of the results and the different reference ranges used for these two analytes. Laboratory medicine is working to obtain more satisfactory and replicable results for this biological marker which is nowadays widely used and appears to have a promising role in different common inflammatory pathologies.

## Data Availability

The original data presented in the study are included in the manuscript. Further requests should be addressed to the corresponding author.
